# The lifestyle factors of physical activity and diet balance associated with HPV infection in China: The cross-sectional study

**DOI:** 10.3389/fonc.2022.1043937

**Published:** 2022-12-07

**Authors:** Yantao Li, Mengping Liu, Peng Huang, Wenxiang Wang, Yuxin Jiang, Zhongzhou Yang, Anli Wang

**Affiliations:** ^1^ College of Sports Medicine and Rehabilitation, Faculty of Sport Rehabilitation, Beijing Sport University, Beijing, China; ^2^ School of Pharmacy, Nanchang University, Nanchang, China; ^3^ Department of Anesthesiology, The Second Affiliated Hospital of Nanchang University, Nanchang, China; ^4^ Department of Gynecology, The First Affiliated Hospital of Nanjing Medical University, Nanjing, China; ^5^ Key Laboratory of Marine Drugs, School of Medicine and Pharmacy, Ocean University of China, Qingdao, China

**Keywords:** diet balance, physical activity, eHealth platform, HPV infection, lifestyle factors

## Abstract

**Background:**

Human wellbeing has been linked with lifestyle factors such as physical activity, diet balance, sleep quality, depression, and anxiety. However, few studies illustrate the relationship between such lifestyle factors and HPV infection. In this study, we investigated the association between lifestyle factors, age, disease status and HPV infection.

**Participants and methods:**

Participants were recruited through a digital eHealth platform in Shenzhen, Mainland China. Both lifestyle factors and cervicovaginal mucus (CVM) samples to test for HPV outcomes were collected from each participant as a cross-sectional study. In addition, the eHealth platform recorded age and current or history diseases, which were adjusted to apply for both univariable and multivariable logistic regression. Furthermore, lifestyle factors were categorized as different levels to conduct stratification analysis.

**Results:**

We recruited 149 HPV positive and 346 HPV negative participants through HPV detection. Physical activity and diet balance were significantly associated with HPV infection in lifestyle factors (P values < 0.001) after adjusting for age and current or history diseases. However, stratified analysis showed three factors were insignificant for HPV infection – namely, sleep quality, depression, and anxiety. Most HPV infections involved a sole HPV serotype (83%), and diet balance was the most significant difference between sole and multiple HPV infections.

**Conclusions:**

Among lifestyle factors, physical inactivity or diet imbalance can significantly increase HPV infection risk. In particular, diet balance might be related to the number of HPV serotypes. Our results suggest that exercising and regulating diet may reduce the risk of HPV infection.

## Introduction

Lifestyle factors is important for both population and individual level to achieve the optimal health, appropriate bodyweight and reducing the gynecological cancer burden in global (1, 2). They include physical activity, diet balance, sleep quality, depression and anxiety impact personal wellness (3). The current prevalence of physical inactivity is estimated to be 23% (4, 5). Hence, the World Health Organization has recommended a 10% increase in physical activity by 2020 (5). Similar with cardiovascular diseases, the physical activity might substantially prevent the overall cancer burden (6).

Though epidemiological research has shown that a diet balance is associated with decreasing cancer mortality (7, 8), related randomized controlled trials (RCT) for diet balance have not unveiled any demonstrative associations (3). This obvious paradox has led to increased targeting of diet balance patterns as a risk factor. In addition, three other risk factors that indicate a plausibly precancerous condition are sleep quality and psychosocial variables such as depression and anxiety (9).

However, relatively few studies have focused on connecting these lifestyle factors with HPV infection or HPV persistent infections, which is the common cause for most cervical cancers (10). HPV infection has been shown to be influenced by age, sexually transmitted diseases (STDs), and tumors. Existing studies have explored the link between HPV and four diseases including anemia (11), endocrine disease (12), metabolic disease (13, 14) and mental disorders (15). They also cover HPV and three diseases in medical history – namely, reproductive tract infection (16, 17), tumors, and consanguinity tumors.

To clearly elucidates the hypothesis between lifestyle factors and HPV infection, we collected the standard questionnaires of lifestyle factors and adjust for the potential confounders of age and other current or history diseases like STDs. The possible mechanisms might monocyte engagement or cell immunity for physical activity (18–20). In diet balance terms, the potential mechanisms might be accounted by vitamin A, lycopene or cell immunity system (21, 22).

## Methods

### Study design and inclusion criteria

This study was approved by the ethics committee of the Institutional Review Board at Beijing Sports University. From May 2021 to August 2021, study population were registered through the digital eHealth platform from Shenzhen in Mainland China, as a cross-sectional study. The inclusion criteria were nonpregnant and nonlactating women for eligible participants. They were also unvaccinated for HPV but had sexual experience at least once in their lifetime. The enrolled participants have not been tested for HPV in the past 12 months and willing to accept the free SeqHPV (Beijing Genomics Institute, China) testing services. Our team explained the study objectives and obtained signed informed consent from participants to collect questionnaires.

### Sample collection and outcome measures

Once participants enrolled this research, they received a mailed package consisting of a nylon conical brush and graphic user instructions of SeqHPV. Participants were required to collect cervicovaginal mucus (CVM) samples by rotating brush and smear samples onto a Flinders Technology Associates (FTA) card. Participants were provided with a package and free express delivery to send their FTA cards to the laboratory. Personalized online guidance was offered if participants had any queries about sampling or delivery.

After the samples were returned to the laboratory, each FTA card was suspended in 5ml PreservCyt media in order for each self-sample to be sequenced for HPV (seqHPV) testing. The main bioinformatic workflows and pipelines have been described in the literature under the same kit as well as methodology (23). These tests independently reported 16 serotypes in total, with 12 high-risk HPV serotypes (hrHPV) as HPV 16, 18, 31, 33, 35, 39, 45, 51, 52, 56, 58, 59 (24); 2 intermediate risks as HPV 66 and 68 (25); and 2 low risk HPV (lrHPV) as HPV 6 and 11 (26). Participants received a test report online. Doctors also provided further advice to resolve any queries they had about their test.

#### Standard questionnaires and scores

A digital eHealth platform tool was developed on May 2020 to record age, lifestyle factors through the standard questionnaires and strategies, and disease status. Physical activity was monitored through the International Physical Activity Questionnaire (IPAQ) and could be beneficial for cancer control. Diet balance, sleep quality, depression and anxiety were recorded by the Diet Balance Index (DBI), Pittsburgh Sleep Quality Index (PSQI), Patient Depression Questionnaire-9 (PDQ-9), and Generalized Anxiety Disorder 7 (GAD-7) respectively. In addition, the digital eHealth platform provided information on HPV prevention courses *via* weekly live streaming while regularly providing advice for participants.

Physical activity was scored in three groups for low level, moderate level, and high-level. Based on IPAQ in **Supplementary 1** (27), three levels had the risk scores as 15, 10 and 5. Diet balance was scored for Low-Bound Score (LBS) indicates that diet intake was less than standard level. LBS had four risk groups as almost no problem -14 ~ -1, low level of under intake -29 ~ -14, moderate level of under intake -43 ~ -29, and high level of under intake -72 ~ -43 (DBI in **Supplementary 2**). Then, the absolute score values were used to calculate diet balance. Sleep quality was scored in three groups as good sleep quality 0 ~ 7, moderate sleep quality 7 ~ 14, and bad sleep quality 14 ~ 21 (PSQI in **Supplementary 3**). Depression was scored in five groups as minimal depression 0 ~ 4, mild depression 4 ~ 9, moderate depression 9 ~ 14, moderately severe depression 14 ~ 19 severe depression 19 ~ 27 (PHQ-9 in **Supplementary 4**). Anxiety was scored in four groups as minimal anxiety 0 ~ 4, mild anxiety 4 ~ 9, moderate anxiety 9~ 14, and severe anxiety 14 ~ 21 (GAD-7 in **Supplementary 5**).

#### Statistical analysis

The preliminary demographics were reported for two groups – one HPV-positive and the other HPV-negative. For the two groups, we summarized the continuous variables of age and lifestyle factors scores with both mean and standard deviation. Disease statuses were summarized as categorical variables with numbers and percentages. Differences were compared using the Wilcoxon rank sum test for continuous variables (28) and the Pearson χ² test for categorical variables (29). In addition, the statistical power was also calculated for lifestyle factors. In order to study the association between lifestyle factors and HPV outcomes, we applied both univariable and multivariable logistic regression (30). We conducted further stratified analysis for different risk groups among lifestyle factors. The reference group was low risk for each lifestyle behavior factor.

To compare and assess the effect of HPV serotype, we differentiated the three risk groups for HPV infection as hrHPV (high-risk HPV), irHPV (intermediate-risk HPV) and lrHPV (low-risk HPV). We also enumerated the number of serotypes 1 ~ 4 from participants. For one serotype (sole) and 2 ~ 4 serotypes (multiple), physical activity and diet balance scores were tested by the Wilcoxon sum rank. All analysis was conducted utilizing R software (4.0.4) for the Macintosh Operating System (Mac OS).

## Results

### Participant recruitment

In total, 495 participants were recruited *via* our digital eHealth tool with 149 HPV positive and 346 HPV negative women (**Table 1**). Patient features for HPV infection are elaborated in **Table 1**. The average age was 41.37 (SD: 7.86) for HPV positive and the average age for HPV negative was 41.56 (SD: 8.51), indicating no significant difference. Disease status was not statistically related to HPV infection. 28% of HPV positive participants and 24% of HPV negative participants had a history of reproductive tract infection.

**Table 1:** HPV statistics for age, disease status and lifestyle factors.

Risk factors	HPV positive (149)	HPV negative (346)	P-values^1^
Demography			
Age	41.37 ± 7.86	41.56 ± 8.51	0.29
Disease status			
Current Anemia	21 (14.09%)	67 (19.36%)	0.16
History of reproductive tract infection	42 (28.19%)	83 (23.99%)	0.32
Current endocrine disease	12 (8.05%)	23 (6.65%)	0.58
Current Metabolic disease	4 (2.68%)	12 (3.47%)	0.65
History of tumor disease	10 (6.71%)	20 (5.78%)	0.69
History of consanguinity tumor	14 (9.40%)	41 (11.85%)	0.43
Current Mental disorder	2 (1.34%)	3 (0.88%)	0.63
Lifestyle behavior scores (Questionnaires^2^)			
Physical activity risk scores^3^ (IPAQ)	14.36 ± 1.95	12.83 ± 3.42	<0.01^##^
Diet balance risk scores^3^ (DBI-16)	25.44 ± 7.33	17.29 ± 10.42	<0.01^##^
Sleep quality risk scores^3^ (PSQI)	4.38 ± 3.28	4.94 ± 3.04	0.01^#^
Depression risk scores^3^ (PHQ-9)	1.92 ± 3.37	2.27 ± 2.98	0.01^#^
Anxiety risk scores (GAD-7)	2.75 ± 5.53	2.92 ± 5.18	0.11

^1^:Statistical differences; Statistical differences is marked ^#^ for 0.01 ≤ P-values < 0.05, ^##^ for 0.001 ≤ P-values < 0.01, ^2^: IPAQ, International Physical Activity Questionnaires; DBI, Diet Balance Index; PSQI, Pittsburgh Sleep Quality Index; PHQ-9, Patient Depression Questionnaire-9; GAD 7, Generalized Anxiety Disorder 7; ^3^: Power of Physical activity, 0.998; Power of Diet Balance > 0.999; Power of Sleep, 0.593; Power of Depression, 0.330.

Among lifestyle behavior scores, there were statistically significant results for physical activity, diet balance, sleep quality, and depression. According to the cross-sectional study, HPV positive had the higher scores than HPV negative group on physical activities (14.36 vs 12.83) and diet balance (25.44 vs 17.29), which the higher scores indicate the unhealthier lifestyle factors. They have a statistical power of 0.998 and over 0.999 respectively with *P-values* < 0.01. Conversely, another three lower scores denote higher risk on sleep scores (4.38 vs 4.94), depression (1.92 vs 2.27) and anxiety (2.75 vs 2.92). However, their statistical power reduced to 0.593 and 0.330 with *P-value* = 0.01.

### Logistic regression for risk factors

To evaluate the association of risk factors for HPV infection, both univariate and multivariate logistic regression were performed for one demography factor, seven disease status factors, and five lifestyle factors (**Table 2**). For the univariate logistic regression, physical inactivity significantly increased risk by 1.03 (95% CI: 1.02-1.05) and diet imbalance by 1.02 (95% CI: 1.01-1.02) in the left half **Table 2**. For the multivariate logistic regression, the increase was 1.20 (95% CI: 1.10-1.33) for physical inactivity and 1.09 (95% CI: 1.06-1.11) for diet imbalance in the right half **Table 2**, after considering the co-relationship with lifestyle factors.

**Table 2:** Univariate and multivariate logistic regression for all factors.

	Univariate logistic regression			Multivariate logistic regression		
Risk factors	Odds ratio	95% CI	P-values	Odds ratio	95% CI	P-values
Demography	
Age	1.00	1.00 - 1.00	0.792	1.01	0.99-1.04	0.32
Disease status						
Current Anemia	1.08	0.97 - 1.20	0.16	1.48	0.87 - 2.59	0.159
History of reproductive tract infection	0.95	0.87 - 1.05	0.325	0.78	0.50 - 1.23	0.281
Current endocrine disease	0.96	0.82 - 1.12	0.577	0.82	0.40 - 1.79	0.613
Current Metabolic disease	1.05	0.84 - 1.33	0.652	1.61	0.53 - 6.08	0.432
History of tumor disease	0.97	0.82 - 1.14	0.691	0.78	0.35 - 1.84	0.555
History of consanguinity tumor	1.05	0.93 - 1.20	0.427	1.41	0.75 - 2.82	0.302
Current Mental disorder	0.9	0.60 - 1.36	0.629	0.76	0.12 - 5.93	0.768
Lifestyle behavior						
Physical inactivity (IPAQ)	1.03	1.02 - 1.05	< 0.001^###^	1.2	1.10 - 1.33	< 0.001^###^
Diet imbalance (DBI-16)	1.02	1.01 - 1.02	< 0.001^###^	1.09	1.06 - 1.11	< 0.001^###^
Poor Sleep quality (PSQI)	0.99	0.98 - 1.00	0.064	0.92	0.83 - 1.00	0.065
Depression severity (PHQ-9)	0.99	0.98 - 1.01	0.255	1.03	0.92 - 1.15	0.623
Anxiety disorder (GAD-7)	1	0.99 - 1.01	0.751	0.99	0.94 - 1.05	0.765

IPAQ, International Physical Activity Questionnaires; DBI, Diet Balance Index; PSQI, Pittsburgh Sleep Quality Index; PHQ-9, Patient Depression Questionnaire-9; GAD 7, Generalized Anxiety Disorder 7; CI, Confidence Interval. Statistical differences are marked ^###^ for P-values < 0.001.

### Lifestyle factors

As expected, patients with high level of physical activities were less likely to be infected with HPV in comparison to participants with low level of physical activity. This association was significant for *P-values* < 0.01 from univariate logistic regression, and *P-values* < 0.001 from multivariate logistic regression. Though patients with high level had the less likelihood of being infected when compared with the moderate level group, it was not statistically significant for *P-value* = 0.22 from univariate logistic regression, and *P-value* = 0.132 from multivariate logistic regression (**Table 3**). For the diet balance, participants with almost no problem had the less possibility of being infected in comparison to participants with low and moderate and high level of under intake. The low level was significantly different for *P-values* < 0.01 from univariate and multivariate logistic regression. However, moderate and high level of under intake demonstrated no significant association (**Table 3**). Another three lifestyle factors were also tested: sleep quality, depression risk, and anxiety risk. However, they were not statistically significant (**Table 3**).

**Table 3:** Risk of HPV infection in different levels of lifestyle factors from univariate and multivariate logistic regression.

Risk factors	HPV positive	HPV negative	Univariate logistic regression			Multivariate logistic regression		
Total	149	346	OR	95% CI	P-values	OR	95% CI	P-values
Physical activity								
High level	3	38	1.00	NA	NA	1.00	NA	NA
Moderate level	13	74	2.14	0.63-10.22	0.22	2.87	0.80 - 13.66	0.132
Low level	133	234	6.85	2.41-29.83	<0.01^##^	7.44	2.49 - 32.08	<0.001^###^
Diet balance								
Almost no problem	15	163	1.00	NA	NA	1.00	NA	NA
Low level of under intake	129	147	9.41	5.42-17.47	<0.01^##^	9.26	5.25-17.44	<0.01^##^
Moderate level of under intake	4	28	1.59	0.42-4.82	0.47	1.52	0.40-4.67	0.491
High level of under intake	1	8	1.51	0.06 – 9.35	0.73	1.25	0.06-7.80	0.837
Sleep quality								
Good sleep quality	126	284	1.00	NA	NA	1.00	NA	NA
Moderate sleep quality	22	59	0.84	0.49-1.42	0.53	0.7	0.35-1.34	0.288
Bad sleep quality	1	3	0.82	0.03-7.12	0.87	1.03	0.03-18.67	0.986
Depression risk								
Minimal	127	284	1.00	NA	NA	1.00	NA	NA
Mild	18	54	0.75	0.41-1.31	0.32	0.95	0.39-2.22	0.903
Moderate	2	6	0.78	0.10-3.58	0.77	1.84	0.2-12.8	0.552
Moderately severe	1	2	1.19	0.04-14.81	0.9	2.42	0.07-107.53	0.603
Severe depression	1	0	NA	NA	NA	NA	NA	NA
Anxiety risk								
Minimal	119	267	1.00	NA	NA	1.00	NA	NA
Mild	15	44	0.77	0.40-1.41	0.41	0.95	0.43-2.09	0.906
Moderate	3	12	0.58	0.13-1.90	0.4	0.42	0.08-1.68	0.249
Severe	12	23	1.18	0.55-2.41	0.67	0.92	0.31-2.69	0.883

CI, Confidence Interval; NA, Not available, Statistical differences are marked ^#^ for 0.01 ≤ P-values < 0.05.

### Physical activity and diet balance among HPV serotypes

**Figure 1:**
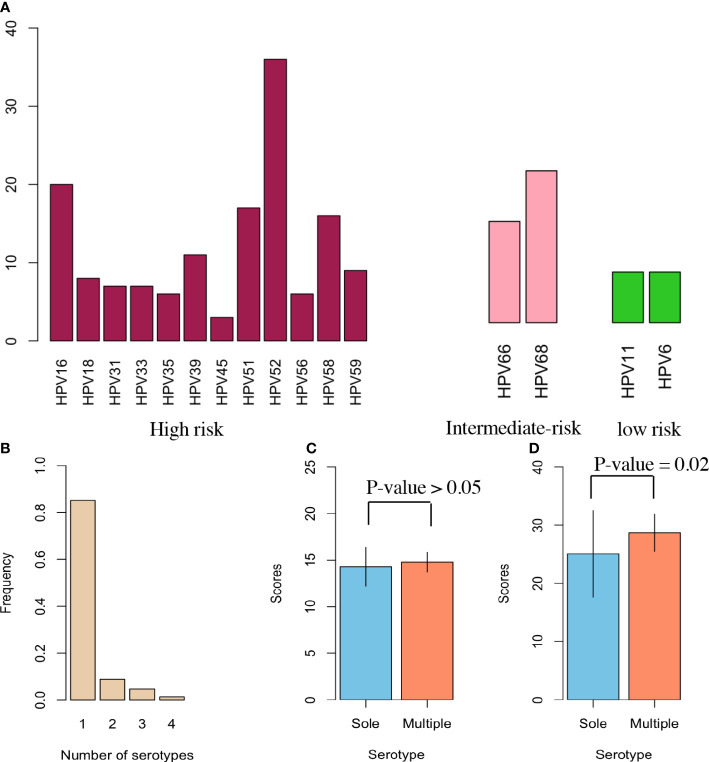
HPV Serotypes in participants. **(A)** Each HPV serotype’s prevalence involved 12 high-risk HPV (hrHPV), 2 intermediate-risk HPV (irHPV), and 2 low-risk HPV (lrHPV). All identifiable serotypes were denoted at the x-axis, and their proportions as percentages were designated at the y-axis among all HPV-infected women. The left red columns indicate the prevalence of each serotype for the 12 hrHPV; the middle pink columns the prevalence of each serotype for the 2 irHPV; the right green columns the prevalence of each serotype for the 2 lrHPV. **(B)** This panel shows the number of serotypes identified for each patient infected with HPV. **(C)** Physical activity risk in two categories of data with sole and multiple serotypes in a bar chart. This plot displays the scores along with mean and SD without illustrating effect size. **(D)** Similar bar chart as **(C)**, but with diet balance risk.

The prevalence of proportions involved 80.66% hrHPVs, 13.81% intermediate HPVs and 5.52% lrHPVs (**Figure 1A**). HPV 52 had the largest prevalence (19.89%) from the hrHPVs, followed by HPV16 (11.05%), then HPV 51 (9.39%). HPV 18 prevalence accounted for 4.42%. For intermediate-risk HPV, HPV68 was more common than HPV66. However, the proportion was the same for HPV 11 (2.21%) and HPV 6 (2.21%) in the lrHPVs group. More than 80% of HPV infections involved one serotype (**Figure 1B**). We then obtained the comparison of two groups with mean and SD for physical activity (**Figure 1C**). A similar comparison was conducted for diet balance and the significance was denoted as *P-values* < 0.05 (**Figure 1D**).

## Discussion

**Figure 2:**
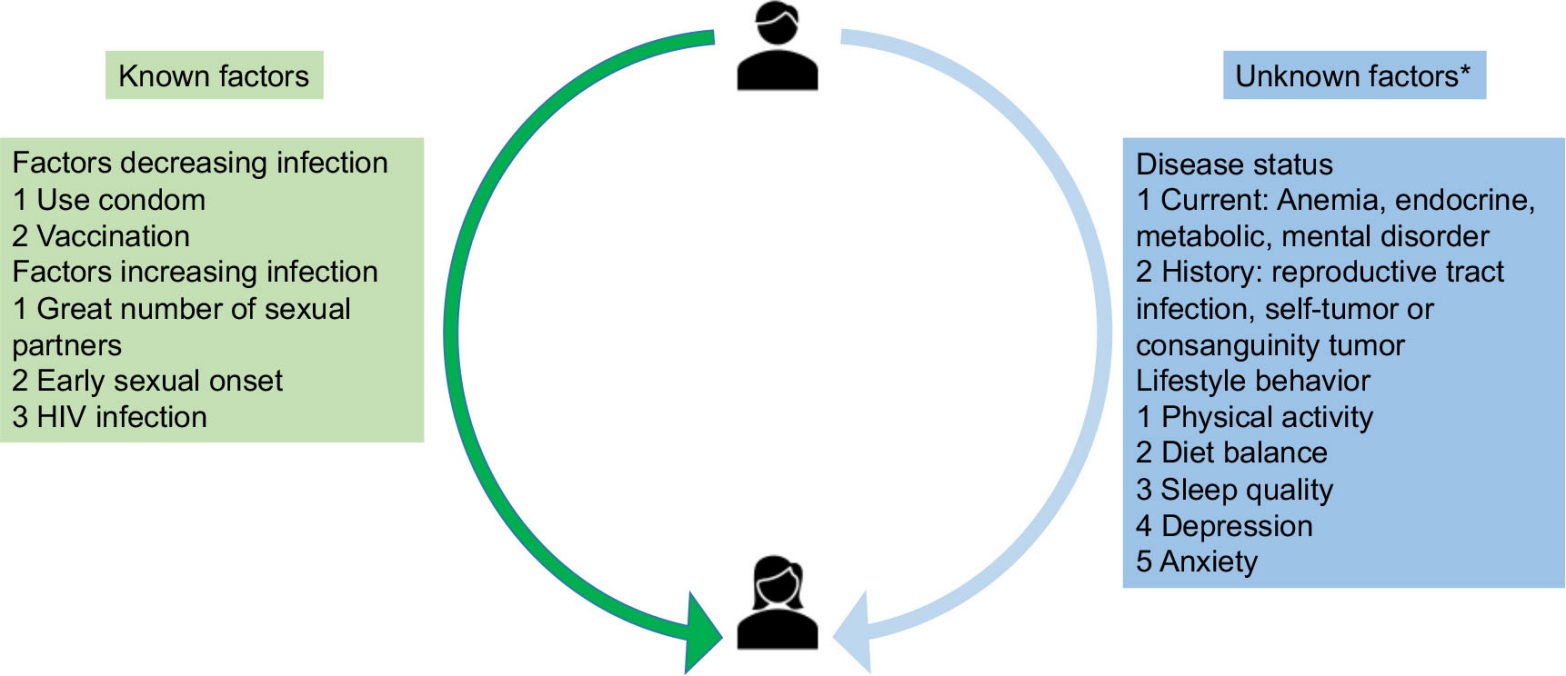
Transmission cycle from male to female and known/unknown factors influencing transmission dynamics HIV, human immunodeficiency virus. *Risk factors for the association direction was unclear before the study in this figure.

Most HPV risk factor studies focus on sexual factors or gynecological infections in women. However, there are few cross-sectional studies that account for lifestyle factors and other current or history diseases infections. In our study, we surveyed 495 participants to investigate whether the lifestyle factors influence transmission dynamics for HPV infection in **Figure 2**. **Figure 2** summarized the known transmission cycle from male to female, and aimed to determine the unknown factors influencing transmission dynamics. Apart from anxiety, four lifestyle factors appeared to demonstrate significant association with HPV infection: physical activity, diet balance, sleep quality, and depression. Meanwhile, current disease or disease history are not significantly correlated with HPV infection.

To adjust for the potential confounders, our pilot study has already summarized 32 clinical factors with finding the possible confounders age as well as history of reproductive tract infection (31). Hence, age, gynecological disease history and current disease status were also collected to adjust for the association. After adjusting for age and disease status, multivariable logistic regression further showed the significant association for physical activity and diet balance (*P-values* < 0.01) among the lifestyle factors.

Our statistical analysis shed light on the most significant lifestyle factors. To decrease HPV infection risk through physical activity, stratification analysis reported the many benefits of high level physical activity. Potential reasons may include a decrease in monocyte engagement in the preinvasive tumor microenvironment (TME) causing a depletion in tumor-associated macrophages (TAMs) (19, 32); or explained by the relation between HPV and human immune system like cell immunity such as CD4/CD8 T cell (20). In other words, we recommend women infected with HPV to undertake higher levels of physical activity. Earlier studies have suggested two possible approaches for increasing physical activity (27). The first involves vigorous intensive activity to achieve a minimum overall physical activity of no less than 1500 metabolic equivalents of task (MET) minutes for at least 3 days per week. The second involves achieving double the amount over the course of an entire week, or 3000 MET minutes every seven days, through any combination of vigorous or moderate intensity activities (which can even include walking).

To further decrease HPV infection risk through diet balance, stratification analysis showed that low level of under intake (OR: 9.41) was much easier to infect HPV than almost no problem diet balance. This may be explained by the reduced intake levels of vitamin A or lycopene for low level of under intake of diet balance *in vivo* (33, 34). Then, both vitamin A or lycopene were further digested through the gastrointestinal tract system activating the relative robust human immune system like cell immunity system (35). Therefore, we recommend two solutions for improving diet balance. Through our questionnaires, one way is to ensure appropriate consumption of dairy products and animal foods consisting of vitamin A. Another way is to intake more fruits (e.g., tomatoes) or vegetables to achieve a stable diet balance (36). Beyond explanations from existing studies, the number of HPV serotypes is another potential justification. There were 12 high-risk HPV (hrHPV) serotypes, 2 intermediate-risk and 2 low-risk serotypes. We found that diet balance scores were significantly increased among those with multiple HPV infections. Thus, the other solution implies that diet balance might be more effective for those with multiple HPV genotypes.

After adjusting for confounders, three lifestyle factors (sleep quality, depression, and anxiety) were shown to be insignificant in relation to HPV infection based on univariable and multivariable logistic regression. Moreover, HPV infection does not appear to be associated with 7 current or history diseases, whether current or history, as validated by univariable (*P-values*: 0.160 ~ 0.652) and multivariable logistic regression (*P-values*: 0.159 ~ 0.768) based on data collected from our eHealth platform.

Beyond lifestyle factors, our study also highlighted the benefits of communication *via* an eHealth platform. On our eHealth platform, participants actively responded to HPV advice. Through the platform, doctors provided weekly livestream training modules with advice about HPV self-sampling and cervical cancer prevention courses. During these sessions, participants were informed about the objective of our study and possible health benefits. In our previous pilot study in May 2020, our team established the eHealth platform and continues to serve HPV high-risk cohorts (37). This regular training led to a high response rate from 2020 to 2021, as reflected by the growing audience size at our weekly livestream. Connecting with a wider audience has made it possible for us to plan further studies. Further ahead, we plan to enlarge the more widespread clinical variables considering the sexual factors. After that, the HPV infection model will be developed to predict the infection period through survival analysis.

### Strengths

In our study, one strength was the age range. The age range of HPV positive participants (41.37 ± 7.86) was comparable with HPV negative participants (41.56 ± 8.51). This age range is also the one in which women experience a high risk for HPV infection. Another strength was how the digital eHealth platform enabled participants to fill in their information electronically *via* their mobile phone. This substantially decreases the scope of human-made error. A third strength is how this study systematically considers the correlation between daily lifestyle factors and HPV infection after adjusting for the impact of age and current diseases or disease history. The final strength is that our study is almost the first article to incorporate five lifestyle factors through the standard questionnaires for HPV infection study in global. Even though other regions have studied some lifestyle factors of HPV infection, like East China (38), UK (39), Brazil (40) and Australia (41), our study is relatively more comprehensive about lifestyle factors of HPV infection till now.

### Weaknesses

There are three limitations to our study. First, other lifestyle factors might affect common HPV clearance risk, such as sexual behavior, smoking, or alcohol consumption. However, our study focused on determining whether the lifestyle factors belong to HPV infection factors. Other studies have shown that HPV infection factors are different from clearance factors. To response the clearance factors, we have already designed the prospective follow-up study systematically. Second, our study locations consisted of more well-off areas such as Shenzhen, where there is greater opportunity for abundant physical activity and dietary resources. A more comprehensive study would expand to other locations such as second-line cities or even the countryside in order to reduce geographical bias. To address this issue, our next formal study will focus on areas that encompass a broader scale, from less economically developed to more economically developed.

## Conclusions

This study suggests that both physical activity and diet balance are significantly beneficial lifestyle factors to reduce HPV infection risk. However, our current evidences showed there were no significant association for another three lifestyle factors as sleep quality, depression, and anxiety. Though we recommend that regular exercise and regulating one’s diet balance might reduce the risk of HPV infection, it still requires the further evidence to intervene HPV persistent infection or further disease progression through the related randomized controlled trials.

## Data availability statement

The original contributions presented in the study are included in the article/**Supplementary Material**. Further inquiries can be directed to the corresponding authors.

## Ethics statement

The studies involving human participants were reviewed and approved by Beijing Sports University (2021173H). The patients/participants provided their written informed consent to participate in this study.

## Author contributions

YL and ZY designed the project and carried out this study. AW found this project. ZY and ML analyzed the data and prepared the manuscript. YJ helped to draft the manuscript and provided the substantial suggestions to improve the manuscript. In addition, WW revised the manuscript together. YL collected the dataset and AW helped to obtain approval from the relevant ethics committees. All authors contributed to the article and approved the submitted version.
